# Reply to: Predictors of coronary artery disease in cardiac arrest survivors: coronary angiography for everyone? A single-center retrospective analysis

**DOI:** 10.5935/0103-507X.20220031-en

**Published:** 2022

**Authors:** Joana Rigueira, Inês Aguiar-Ricardo, Pedro Carrilho-Ferreira, Miguel Nobre Menezes, Sara Pereira, Pedro S. Morais, Pedro Canas da Silva, Fausto J. Pinto

**Affiliations:** 1 Cardiology Service, Centro Hospitalar Universitário Lisboa Norte, Faculdade de Medicina, Universidade de Lisboa - Lisboa, Portugal.

**TO THE EDITOR**

We thank doctors Barriuso, Irigaray, Rivera and Fernández-Rodríguez for their interest in our paper entitled “Predictors of coronary artery disease in cardiac arrest survivors: coronary angiography for everyone? A single-center retrospective analysis”, which aimed to identify predictors of coronary artery disease in survivors of cardiac arrest (CA), to define the best timing for coronary angiography, and to establish the relationship between coronary artery disease (CAD) and mortality.^(1)^

The authors of this letter raised three important questions regarding our paper. First, regarding the definition of significant CAD being based solely on angiographic assessments. In our paper, the cutoffs for significant CAD were a lumen reduction of at least 50% in the left main artery and of at least 70% in the remaining vessels, as recommended in the recently published American Heart Association (AHA) guidelines.^(2)^ In our center, we routinely perform multiple angiographic views to reduce the error of visual estimation of the degree of coronary stenosis. However, we understand the authors’ point, and herein, it is important to differentiate the concept of intermediate stenosis that might not cause ischemia and unstable plaque that might be the culprit for the event.

Intermediate stenosis is classically defined by a lumen reduction of 40 - 69%^(2)^ in non-left main vessels, although a higher range can be found in some studies. To evaluate whether such lesions cause ischemia, a noninvasive stress test or invasive evaluation with fractional flow reserve (FFR) or instantaneous wave-free ratio (iFR) is recommended. However, recent CA is usually a contraindication for noninvasive stress tests. With regard to coronary physiology, there is very limited evidence for FFR, iFR or other indices in this clinical context, mainly because unstable patients (such as patients after CA) have been excluded from the main trials assessing these techniques, while most studies have focused on chronic coronary syndrome or non-culprit lesions of acute coronary syndrome (ACS), such as in the iFR-Swedeheart trial.^(3)^ In addition, there are no strong data to suggest that FFR guidance improves clinical outcomes in ACS. In fact, there is some evidence suggesting that microvascular dysfunction during ACS might compromise the achievement of maximal hyperemia, increasing the number of false negatives with FFR.^(4)^ Additionally, the recent FLOWER-MI study,^(5)^ the only trial directly comparing FFR *versus* angiography for non-culprit lesions in the ACS setting, did not show superiority of an FFR-based strategy. Thus, there is no evidence that supports invasive physiology to identify the culprit lesion in ACS or after CA.

The discussion on stable and unstable plaques is very interesting, as there is some evidence that only percutaneous intervention (PCI) of unstable lesions might be beneficial in patients after CA.^(6)^ Importantly, if operators found a clear culprit lesion by angiographic criteria, these patients were considered to have significant coronary artery disease in our study - perhaps that was not made sufficiently clear. Regarding intracoronary imaging, there were patients in our sample in which intravascular ultrasound (IVUS) and optical coherence tomography (OCT) were used according to the operator’s choice, namely, in patients with type 4 myocardial infarction and to plan and optimize PCI results. However, as this was not the purpose of our paper, these data were not reviewed in detail. There was a recently published paper^(7)^ based on a multicenter myocardial infarction registry that supports the utilization of IVUS to reduce major adverse cardiovascular events in ACS; however, death (the outcome evaluated in our study) was not reduced. In summary, the evidence to support the routine use of IVUS and OCT to guide primary PCI in patients after CA or ACS is very scarce, but it is certainly an important field of research and one where we are likely to learn more in the coming years from currently ongoing trials.

Importantly, both physiological and intracoronary imaging require wiring coronary vessels, and OCT requires additional contrast. Both of these add further complexity, risk and length to an already unstable clinical setting, and one cannot rule out that such disadvantages may negatively affect prognosis. The simplicity and very low risk of coronary angiography may be a better option than upscaling invasive modalities, as we all (especially interventionalists) often learn the hard way.

Second, regarding defining the cause of CA, as mentioned in our paper,^(1)^ the etiology of CA is often difficult to ascertain in an emergency setting, as medical history is often unavailable and exam results are difficult to interpret, with abnormalities in electrocardiogram, echo and troponin values not always caused by CAD. In our sample, only ST-segment elevation and the presence of wall motion abnormalities (WMA) were independent predictors of coronary artery disease, and even in these subgroups of patients, 12% of patients with ST-elevation and 7% with WMA did not have significant CAD. Therefore, we agree that better methods to diagnose the cause of CA are needed to improve the management of these patients and select the most appropriate therapies while avoiding unnecessary ones. The development of shorter cardiac magnetic resonance (CMR) protocols and CMR compatible organ support equipment that might be applied to unstable patients, such as the utilization of mapping sequences that allow myocardial characterization without gadolinium, may in the future contribute to a better diagnostic approach in this clinical scenario. However, currently these techniques are not widely available in this clinical context.

Third, regarding the prognostic impact of coronary angiography and PCI in patients after CA, as mentioned in our paper^(1)^ and in the literature, most patients die from neurological complications after CA. Therefore, the development and early implementation of neuroprotective measures is probably the factor with the highest impact on these patients’ survival. Our results are in keeping with this concept and those of recent trials, such as the COACT^(8)^ and TOMAHAWK trials,^(9)^ where immediate angiography provided no benefit in the 30-day risk of death from any cause. Our study is a retrospective analysis with all the limitations associated with this design. We agree that the definition of the cause of death would have been interesting and valuable. However, this was not the purpose of the study, and sometimes it is difficult to infer the cause of death based on clinical records, as these patients might have had long hospital admissions with several complications. All-cause death was therefore the only fully objective endpoint we could have selected, as we aimed to avoid the risk of significant bias that any retrospective analysis might contain.

In conclusion, we agree that better and more reliable diagnostic tools are needed to establish the cause of CA in order to select patients who will benefit the most from coronary angiography, identifying which coronary lesions may benefit from revascularization leading to prognostic improvements in such challenging patients. We hope that our real-world study has made a valuable contribution to the field.
